# Sexual Well-Being in Individuals with Schizophrenia: A Pilot Study on the Role of Self-Esteem and Acceptance of Illness

**DOI:** 10.3390/ejihpe13070097

**Published:** 2023-07-19

**Authors:** Rafał Gerymski, Marta Szeląg

**Affiliations:** 1 Department of Health Psychology and Quality of Life, Institute of Psychology, Opole University, 45-040 Opole, Poland; 2 Hospital of the Ministry of Interior and Administration in Opole, 45-075 Opole, Poland; marta.szelag@outlook.com

**Keywords:** schizophrenia, sexual well-being, acceptance of illness, self-esteem, well-being

## Abstract

Schizophrenia is one of the most severe chronic mental illnesses. It drastically changes an individual’s life and well-being. Sexual well-being in schizophrenia is often sidelined, even though it is one of the main areas for maintaining quality of life. Based on the conservation of resources theory (COR) and empirical data, we assume that self-esteem and acceptance of illness help in coping with schizophrenia and maintaining sexual well-being. This pilot study aimed to determine the relationship between self-esteem, acceptance of illness, and sexual well-being in Polish individuals with schizophrenia. The results of 60 individuals were included in this study. In the present study, the Self-Esteem Scale (SES), Acceptance of Illness Scale (AIS), and Short Sexual Well-Being Scale (SSWBS) were used. The study showed a significant association between self-esteem, acceptance of illness, and sexual well-being. Self-esteem (β = 0.62) and acceptance of illness (β = 0.55) acted as positive and significant predictors of sexual well-being in individuals with schizophrenia. Acceptance of illness was also found to play a mediating role between self-esteem and sexual well-being in people with schizophrenia (indirect effect: β = 0.34; *LLCI* = 0.063; *ULCI* = 0.839). The current pilot study demonstrates the relationship between self-esteem, acceptance of illness, and sexual well-being. Our findings highlight the significant role of acceptance of illness in maintaining sexual well-being among individuals with schizophrenia.

## 1. Introduction

Schizophrenia is one of the most severe chronic illnesses in mental health. It is characterized by a diverse clinical image. Patients with a diagnosis of schizophrenia struggle with positive, negative and cognitive symptoms [1]. The course of schizophrenia varies and is unpredictable but, for most patients, the illness is associated with experiencing exacerbations and readmissions to hospitals and periods of remission [2]. Schizophrenia can drastically change an individual’s life and well-being. More severe negative symptoms of schizophrenia correlate with lower psychological well-being and many constructs influencing well-being [3,4]. Additionally, negative, positive and cognitive symptoms are negatively related to the quality of life in schizophrenia [5]. An area that is often overlooked in discussions is the sex lives of individuals with schizophrenia, even though sexual dysfunctions in this group are common and can significantly reduce the sexual well-being of patients [6,7]. Barker and Vigod [8] noted in their review that women with a diagnosis of schizophrenia showed lower sexual needs due to an impaired libido. In addition, both positive and negative symptoms of schizophrenia have been shown to intensify sexual dysfunction and decrease sexual satisfaction [8].

McCann and colleagues [9] also point out that, although in clinical practice the quality of services has improved significantly over recent years, there are still problems in supporting patients with their sexual health. In the face of these reports, it is important to help individuals with schizophrenia maintain their sexual well-being by influencing significant predictors of sexual well-being. Based on the fact that personal resources play an important role in the functioning of individuals with schizophrenia [10,11,12], we decided to investigate the associations between sexual well-being, acceptance of illness and self-esteem, which have a significant impact on intimate life and sexuality in individuals with schizophrenia. 

### 1.1. The Relationship between Self-Esteem and Sexual Well-Being in Schizophrenia

Self-esteem is widely discussed in the literature in the context of mental illnesses [13,14], including among individuals with schizophrenia spectrum disorders [15,16]. Self-esteem appears to have a significant impact on sex life [17] and is defined as the subjective attitude towards the self [18,19]. According to Rosenberg [18], people have different attitudes towards different objects, including themselves. Individuals with high self-esteem have a positive attitude towards themselves and the belief that they are a “good enough” and valuable person. In turn, negative attitudes to self are expressed by negative thinking about oneself and dissatisfaction with oneself and one’s accomplishments. Coopersmith [20] states that self-esteem arises from making evaluations about oneself and is often reflected in attitudes and expressed opinions about oneself. Based on this theoretical perspective, attention is directed towards seeing one’s potential and chances for success, self-confidence and self-respect. Despite different theoretical approaches to the concept of self-esteem, subjective positive evaluation of self can positively affect emotional and physical satisfaction with one’s sexuality and sexual relationships.

Empirical studies mainly show associations between self-esteem and sexual satisfaction, also among individuals with somatic and mental illnesses [17,21,22]. However, sexual well-being cannot be equated with sexual satisfaction [23,24]. Stephenson and Meston [25] consider sexual satisfaction as one component of sexual well-being. Sexual well-being is based on emotional, sexual and cognitive satisfaction with one’s sexuality and sexual relationships, whereas sexual satisfaction only relates to the physical component of human sexuality [23,24,26,27]. Self-esteem is related to many constructs influencing our sexual well-being. Robinson and Cameron [28] showed relationships between self-esteem and satisfaction and commitment in relationships. Individuals with higher self-esteem were found to be more engaged in their relationships and to experience greater relationship satisfaction. Other research indicates that a higher level of self-esteem is associated with a greater ability to communicate about satisfying sexual behaviors in intimate relationships [29]. It is also related to greater sexual assertiveness [30]. Sprecher and Hendrick [31] found that self-esteem is associated with self-disclosure in an intimate relationship. On the other hand, self-disclosure is related to greater well-being [32]. A lower level of self-esteem is also associated with indirectly seeking support from a partner, although this strategy often leads to rejection from the partner [33]. Additionally, Tayebi and colleagues [34] showed that lower self-esteem is associated with higher rates of sexual dysfunction. Also, the direct relationship between self-esteem and sexual well-being has been confirmed in previous studies by Gerymski et al. [24].

### 1.2. The Acceptance of Schizophrenia, Self-Esteem and Sexual Well-Being

The process of adaptation in schizophrenia is extremely difficult for patients, due to the unpredictable course of this illness, its symptoms and pharmacotherapy, which play a significant role during treatment [1]. Acceptance of illness, especially a serious one, is a major challenge for the patient. Because schizophrenia is a chronic illness, it somewhat forces the individual to adapt to a different lifestyle. According to Zauszniewski and colleagues [35], acceptance of illness is defined as an adaptation to other conditions of life, including limitations resulting from the illness. It is also defined as a process during which an individual gradually adapts to a new situation [36,37]. During this process, the person integrates the illness with other characteristics and their lifestyle. According to Rüsch and Corrigan [38], working on acceptance of illness is one element of motivational interviewing, which is a form of help for individuals with schizophrenia. 

Empirical studies show that personal resources play a significant role in the process of adaptation to illness. Studies indicate that self-esteem is associated with acceptance of illness [39,40]. The level of acceptance of the illness is visible in the individual’s daily functioning in life. Simmons and colleagues [41] found that acceptance of illness is related to interpersonal relationships. A lower level of acceptance of the illness is associated with greater problems with social relationships. Individuals who accept their illness are open to the world and people, and do not avoid social encounters. Reconciliation with the illness and acceptance of this situation impacts on health-related quality of life [36,37,42]. In addition, Lysaker and colleagues [43] pointed out that insight into one’s illness is associated with a greater quality of life in schizophrenia spectrum disorders. Based on these theoretical and empirical reports, we can assume that a higher level of acceptance of the illness will also be associated with greater well-being in terms of one’s sexuality and sex life. 

### 1.3. The Potential Indirect Effect

No previous research has investigated the relationship between self-esteem, acceptance of illness and sexual well-being in individuals with schizophrenia. To date, we have found only one study related to this subject, which is focused on a different group of patients. Furmańska et al. [17] studied the relationship between acceptance of illness, self-esteem, sexual satisfaction, level of disability, duration of treatment and time of diagnosis in patients with multiple sclerosis. They found that acceptance of illness was not related to self-esteem and sexual satisfaction in the studied group of individuals, but showed that self-esteem was moderately related to sexual satisfaction in multiple sclerosis patients. This is a single piece of evidence for a relationship between the mentioned variables, and therefore, should be further investigated. Moreover, this study does not address self-esteem, acceptance of illness and sexual well-being in individuals with schizophrenia. Many personal resources impact one’s sexuality, sex life and intimate relationships, for instance: personality traits, self-acceptance and self-esteem [17,24,29,44]. All these psychological variables also support the process of adaptation to the situation of illness. Therefore, we can assume that self-esteem does not directly affect sexual well-being, but through the mediation of acceptance of the illness. Acceptance of illness leads to minimizing the impact of the illness on life and favors greater sexual well-being. This hypothesis is also supported by the conservation of resources theory (COR), which states that resources facilitate survival or enable the acquisition of others that help in coping with a critical life situation. In the situation of struggling with schizophrenia, acceptance of the illness increases coping effectiveness, reduces distress, and thus contributes to maintaining well-being in the sphere of sexuality and sex life. Previous studies have also shown that acceptance of illness plays a mediating role between some variables related to self-esteem and sexual well-being. For example, acceptance of illness mediates the relationship between cancer-related complaints and psychological distresses [45]. The mediating role of acceptance of illness is also significant in the relationship between neurological disability and quality of life and its two spheres—the physical sphere and the mental sphere [36].

### 1.4. The Aim of This Study

**Figure 1:**
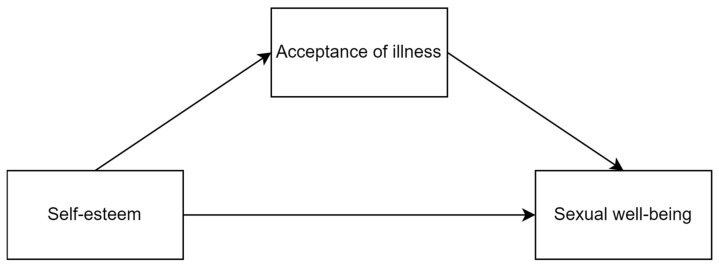
The visualization of the hypothesized mediation model.

This pilot study aimed to extend previous research on the relationship between self-esteem, acceptance of illness and sexual well-being. It is one of the first studies to verify the relationship between those three variables in individuals with schizophrenia and to assess a new measurement of sexual well-being (the Short Sexual Well-Being Scale) in this group of people. Additionally, it is the first to verify the role of the acceptance of illness in the sexual sphere. Based on prior studies, we formulated the following hypotheses: (1) self-esteem is positively related to acceptance of illness, (2) both self-esteem and acceptance of illness are positively related to sexual well-being and (3) acceptance of illness is a mediator in the relationship between self-esteem and sexual well-being (see Figure 1). 

## 2. Materials and Methods

### 2.1. Participants and Procedure

The presented pilot study participants were recruited through support groups for people diagnosed with schizophrenia in Poland. All study participants were invited to participate in this study based on an online invitation. Those who were willing to participate, applied to us based on the contact information contained in the invitation. Inclusion criteria were the following: (1) having a diagnosis in the range of F20.0–F20.9 according to the ICD-10 diagnostic manual, (2) being at least 18 years old, (3) being a person whose mother tongue is Polish and (4) having the ability to give informed consent. Exclusion criteria were the following: (1) not attending psychiatrist consultations, (2) being hospitalized in the last 30 days and (3) inability to fill out a questionnaire for any reason. The questionnaires were delivered to the individuals by a trained study assistant. The study participants were informed about the anonymity of the study. They could stop filling out the questionnaires at any time and without giving any reason. All respondents gave verbal and on-paper (by answering the question: *Do you consent to participate in the study?*) consent to participate in this study. 

Initially, 64 individuals were recruited to participate in the study. However, four individuals were excluded based on the inclusion and exclusion criteria. Therefore, 60 Polish individuals with schizophrenia took part in this study, including 31 women and 29 men, aged between 18 and 58 years (*M* = 31.27; *SD* = 9.04). More detailed information is available in Table 1.

### 2.2. Measures

Three scales were used in the present pilot study. Well-known and broadly available Rosenberg’s Self-Esteem Scale (SES) was used. SES is a 10-item scale on a 4-point answer scale, ranging from 1 (I definitely agree) to 4 (I definitely disagree). It measures self-esteem with items such as: *I feel that I have a number of good qualities*. The SES measures a global level of self-esteem, without any subscales. In this scale, a higher SES score means higher self-esteem [18]. In the present study, SES was characterized by good reliability (Cronbach’s alpha = 0.89).

Additionally, the Acceptance of Illness Scale (AIS). It is an 8-item scale on a 5-point answer scale (1—I definitely agree; 5—I definitely disagree). It measures one’s attitude towards illness with items such as: *I have a hard time adjusting to the limitations of my illness.* A higher score on the scale indicates a higher level of illness acceptance. The AIS does not have any subscales [46]. In the present study, AIS was characterized by good reliability (Cronbach’s alpha = 0.85).

Short Sexual Well-Being Scale (SSWBS) contains five items measuring the level of sexual well-being. Two versions of the scale are available: 4-point and 7-point versions. In this study, the 7-point version of the scale was used (where 1—I completely disagree; 7—I completely agree). It measures sexual well-being with items such as: *I consider myself sexually fulfilled.* A higher score on the scale indicates a higher level of sexual well-being. The SSWBS does not have any subscales [23]. In the present study, SSWBS was characterized by good reliability (Cronbach’s alpha = 0.85).

### 2.3. Statistical Analysis

Parametric analyzes were chosen to analyze the data for several reasons: (1) the skewness values of the tested variables ranged from 0.36 to 0.43 and the kurtosis values of the tested variables ranged from −1.07 to −0.96 (which indicates the low asymmetry of the studied variables); (2) the tested subgroups were equal; (3) parametric analyzes have more structured recommendations on effect size measures; and (4) parametric tests have high statistical power and resistance to breaking their assumptions (e.g., on distribution and homogeneity of variance). Therefore, the group differences were calculated with the *t*-test analysis with the Cohen’s d effect size measure. The significance of the relationships was tested with Pearson’s r correlation. Mediation analysis was performed using PROCESS v3.4 [47] with additional R ^2^ and f ^2^ effect size measures. All results were analyzed using IBM SPSS Statistics 28 (Predictive Solutions Sp. z o.o., Cracow, Poland). Power analysis was conducted using G*Power 3.1.9.7 [48] and Monte Carlo simulation [49]. A significance level of α = 0.05 was adopted as the threshold value for statistical significance.

## 3. Results

### 3.1. Group Differences

Before verifying the mediation hypothesis, we decided to check whether the tested sample of individuals with schizophrenia is a homogeneous group. For this purpose, the *t*-test analysis was used. We decided to use two variables as the grouping ones: gender and attending psychologist consultations. Other grouping variables were not taken into account due to the small size of most of the available categories (see Table 1 for reference). The analysis showed only one significant difference: men had significantly higher self-esteem than women (moderate effect size). Due to the lack of strong effect sizes and non-significant results in five out of six comparisons, we decided to treat the present group of study participants as homogeneous. More detailed data are shown in Table 2.

### 3.2. Correlations

In the second step of the statistical analysis, we decided to check whether the tested variables were significantly related. Pearson’s r correlation showed that self-esteem was positively related to acceptance of illness (large effect size) and sexual well-being (moderate effect size). Acceptance of illness was also positively related to life satisfaction (moderate effect size). More detailed data are shown in Table 3.

### 3.3. Mediation Analysis

In the next step, we decided to perform a mediation analysis due to the significant relationships found in the Pearson’s r correlation analysis and presented theoretical background. According to Creedon and Hayes [50], the obtained number of respondents (*n* = 60) allows us to carry out the mediation analysis. Therefore, the bootstrapping mediation using the PROCESS macro was used with the declared number of 5000 samples [51]. The analysis was performed using model number 4 [47]. 

**Figure 2:**
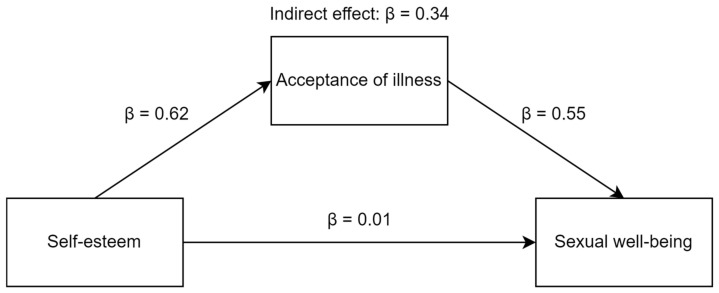
Standardized Beta coefficients of the obtained model.

The PROCESS macro results showed that self-esteem was not a direct predictor of study participants’ sexual well-being. On the other hand, acceptance of illness served as a significant and positive predictor of the sexual well-being of the studied individuals with schizophrenia. Analysis of the confidence intervals of the indirect effect suggested that acceptance of illness served as the mediator in the relationship between self-esteem and sexual well-being in the studied sample. More detailed data are shown in Figure 2 and Table 4.

### 3.4. Sensitivity Power Analysis

The sensitivity power analysis performed using G*Power 3.1.9.7 [48] suggested that, for the power levels of 1-Beta = 0.80 and a significance of α = 0.05, the obtained sample (*n* = 60) was sufficient to detect effects larger than rho = 0.31 and *f*^2^ = 0.11. In addition, the Monte Carlo simulation [49] with a number of 5000 replications and 20,000 Monte Carlo draws was performed at a confidence level of 95%. The simulation confirmed that the presented indirect effect can be interpreted due to sufficient statistical power. Therefore, it can be concluded that the presented significant results of the pilot study can be discussed.

## 4. Discussion

The purpose of the present pilot study was to verify the role of acceptance of illness as the mediator of the relationship between self-esteem and sexual well-being. Our research found associations between these variables and revealed that acceptance of illness significantly mediated the relationship between self-esteem and sexual well-being in individuals with schizophrenia.

### 4.1. Possible Explanations and Clinical Implications of the Presented Results

Firstly, the homogeneity of the examined sample of individuals with schizophrenia was verified. The analysis showed that men with schizophrenia had higher self-esteem than women with schizophrenia. This result may be due to the experience of greater difficulty in coping with the schizophrenia spectrum disorder among women. In a review of studies on gender differences in schizophrenia, Falkenburg and Tracy [52] noted that positive and cognitive symptoms occur more frequently and with greater intensity in women. The unfavorable course of the illness in women may contribute to their lower self-esteem. Additionally, women tend to worry more than men [53]. In turn, greater worry among individuals with schizophrenia spectrum disorders is associated with worse social functioning [54].

Schizophrenia is a severe, chronic illness that affects a person’s level of satisfaction in various areas. Therefore, we investigated the relationship between self-esteem, acceptance of illness and sexual well-being. The correlation results demonstrated that self-esteem was significantly associated with acceptance of illness. Having a positive attitude towards oneself was related to acceptance of the difficulties and limitations associated with schizophrenia. This result confirms the first hypothesis and is in line with previous studies indicating significant relationships between self-esteem and acceptance of illness [17,39]. In addition, self-esteem and acceptance of illness appeared to be associated with sexual well-being, although the strength of this association was moderate. Our research extends previous findings. A particularly interesting result is the relationship between acceptance of illness and sexual well-being. Previous studies have mainly focused on investigating the relationship between acceptance of illness, well-being and quality of life [55,56]. Our study shows that acceptance of one’s illness is also related to physical, emotional and social well-being in terms of a person’s sexuality and satisfaction with the quality of sexual relationships.

In our study, we also hypothesized that the relationship between self-esteem and sexual well-being would be mediated by acceptance of illness. The analysis revealed that acceptance of illness was a significant mediator in the relationship between self-esteem and sexual well-being in individuals with schizophrenia. Self-esteem is positively associated with acceptance of illness which, in turn, favors the fact that the sampled individuals with schizophrenia experienced sexual well-being. The present study thus contributes to previous knowledge by demonstrating that, although there are direct associations between self-esteem and sexual well-being in individuals with schizophrenia, indirect associations, in which illness acceptance was the mediating variable, are stronger. This central result in our study is in line with the researchers’ view that the quality of sexual life depends on many factors that interact with each other [8,23,24,27]. Based on our knowledge, this is the first study in individuals with schizophrenia to show the mediational role of acceptance of illness in the relationship between self-esteem and sexual well-being.

By testing the mediation effects, our study sheds new light on the interplay of personal resources in the sexual well-being of patients with schizophrenia, which also provides practical implications for clinicians. Our results indicate the significant role of acceptance of illness in maintaining satisfaction with one’s sexuality and sexual relationships among individuals with schizophrenia. It is important not to overlook the sex life of individuals with schizophrenia, as it is one of the main areas for maintaining well-being and quality of life [23,25,26]. The authors of this study hope to encourage clinicians in their practice to work with patients with schizophrenia based on the adaptation and acceptance of their new and demanding life situation and the limitations of their illness.

### 4.2. Limitations

The present study verified the role of self-esteem and acceptance of illness in shaping well-being in individuals with schizophrenia. According to the authors’ knowledge, it is one of the first to discuss the concept of sexual well-being from the perspective of schizophrenia. Unfortunately, like any research project, this one is not free from limitations. Firstly, our results are based on a cross-sectional study. The mediational model does not allow us to draw any causal conclusions regarding the observed relationships. Although the opposite causal direction can occur, our model based on the theoretical background of COR [57] validates the current findings without any definite causal statements. Longitudinal research would be needed to determine the final causality. Secondly, the sample was treated in the analyses as a homogeneous group. This does not mean, however, that all respondents were very similar to each other. Only one aspect of sexuality was assessed, i.e., sexual well-being. The omission of testing for the occurrence of sexual dysfunction caused by a health condition or other sexuality-related variables is a major limitation of the presented research project and should be taken into account in future studies. Thirdly, we did not verify the role of prescribed medication in the sexual well-being of studied individuals with schizophrenia. The study participants took different medications, in different doses and configurations. Despite consulting our results with a team of psychiatrists, we were unable to draw coherent conclusions from these data. Lastly, the presented pilot study shows the results based on 60 individuals with schizophrenia. Despite positive results gathered via various power analyses, the tested relationships should be tested on a larger, representative sample to reinforce the conclusions of this study.

## 5. Conclusions

The well-being of one’s sexuality, sex life and intimate relationships is a fundamental human right and need. To our knowledge, this is the first study performed among individuals with schizophrenia to indicate the mediating role of acceptance of illness in the relationship between self-esteem and sexual well-being. Self-esteem is positively associated with acceptance of illness which, in turn, favors the fact that the sampled individuals with schizophrenia experienced sexual well-being. Our findings highlight the significant role of acceptance of illness in maintaining satisfying sexual health among patients with schizophrenia and thus encourage clinicians to work with patients based on acceptance of the diagnosis of schizophrenia and the limitations resulting from this illness. Our results are particularly important for Polish practitioners. There are no general clinical interview guidelines in Poland, and the scope of its content varies from institution to institution. The interview questions asked before admission to the hospitals’ wards and psychological centers usually do not include questions about one’s sexual sphere, or are just simply related to one’s sexual activity (assessed based on one sentence: *Are you sexually active?*). The Mental Health Centers Pilot (in Polish: *Pilotaż Centrów Zdrowia Psychicznego*) is a Polish Ministry of Health’s program, the aim of which is to ensure free 24 h psychological support, and provide a diagnostic and therapeutic standard [58]. Unfortunately, its Diagnostic and Therapeutic Standards Draft (in Polish: *Projekt Standardu Diagnostyczno-Terapeutycznego*) does not include questions about one’s sexual sphere. Our study draws attention to the fact that the sexual sphere is significantly related to the acceptance of illness in individuals with schizophrenia, so it should also be taken into account when working with those individuals and assessing their well-being.

## Figures and Tables

**Table 1:** Characteristics of the studied sample.

	***M***	***SD***	***Min***	***Max***
**Age** (all participants)	31.27	9.04	18.00	58.00
**Age** (women)	30.09	8.67	18.00	46.00
**Age** (men)	32.52	9.41	18.00	58.00
**Time of diagnosis** (number of years back)	7.98	6.82	1.00	30.00
	***n***		**%**	
**Gender**				
Women	31		51.67%	
Men	29		48.33%	
**Education**				
Basic	7		11.67%	
Vocational	1		1.67%	
Secondary	29		48.33%	
Higher	23		38.33%	
**Marital Status**				
Single	41		68.33%	
In a relationship	7		11.67%	
Married	12		20.00%	
**Place of Residence**				
Village	14		23.33%	
Town/city	46		76.67%	
**Professional activity**				
Unemployed	7		11.67%	
Employed	29		48.33%	
Student	8		13.33%	
Pensioner	16		26.67%	
**Attending psychiatrist consultations**				
Yes	60		100.00%	
No	0		0.00%	
**Attending psychologist consultations**				
Yes	30		50.00%	
No	30		50.00%	

*Note*: *M*—mean; *SD*—standard deviation; *Min*—minimum; *Max*—maximum.

**Table 2:** Results of the *t*-test analysis.

	**Gender**						***t* (*df*)**	***p***	***LLCI***	***ULCI***	***d***
	**Women**			**Men**							
	**M**	**SD**	***n***	***M***	***SD***	***n***					
Self-esteem	22.48	8.59	31	27.14	7.93	29	−2.18 (58)	0.034	−8.933	−0.375	0.56
Acceptance of illness	22.00	10.50		22.48	10.35		−0.18 (58)	0.858	−5.876	4.911	0.05
Sexual well-being	16.00	8.85		14.62	9.17		0.59 (58)	0.556	−3.278	6.037	0.15
	**Attending Consultations with a Psychologist**						***t* (*df*)**	***p***	***LLCI***	***ULCI***	***d***
	**Yes**			**No**							
	***M***	***SD***	***n***	***M***	***SD***	***n***					
Self-esteem	24.37	8.83	30	25.10	8.36	30	−0.33 (58)	0.742	−5.177	3.710	0.09
Acceptance of illness	22.63	10.13		21.83	10.72		0.30 (58)	0.767	−4.588	6.188	0.08
Sexual well-being	15.93	8.98		14.73	9.05		0.52 (58)	0.608	−3.458	5.858	0.13

*Note*: *M*—mean; *SD*—standard deviation; *n*—number of participants in a subgroup; *t*—*t*-test statistic; *df*—degrees of freedom; *p*—probability value; *LLCI*—lower bound confidence interval; *ULCI*—upper bound confidence interval; *d*—Cohen’s effect size measure for two means comparisons.

**Table 3:** Results of the Pearson’s r correlation analysis.

	α	*M*	*SD*	1.	2.	3.
1. Self-esteem	0.89	24.73	8.53	-		
2. Acceptance of illness	0.93	22.23	10.34	0.62 ***	-	
3. Sexual well-being	0.85	15.33	8.96	0.34 **	0.55 ***	-

*Note*: ** *p* < 0.01; *** *p* < 0.001; α—Cronbach’s alpha (reliability coefficient); *M*—mean; *SD*—standard deviation; kurtosis ranged from −1.07 to −0.96; skewness ranged from 0.36 to 0.43.

**Table 4:** Results of the PROCESS Model 4 analysis.

Path			Symbol	β	*SE*	*LLCI*	*ULCI*	*R* ^2^	*f* ^2^
*X*	***→***	*M*	*a*	0.62	0.12	0.501	1.001	0.38	0.61
*M*	***→***	*Y*	*b*	0.55	0.15	0.293	0.299	0.30	0.43
*X*	***→***	*Y*	*c′*	0.01	0.12	−0.231	0.719		
Total effect			*c*	0.34	0.13	0.101	0.620	-	-
Indirect effect			a*b	0.34	0.20	0.063	0.839	-	-

*Note: X*—self-esteem; *M*—acceptance of illness; *Y*—sexual well-being; β—*Beta*, the standardized regression coefficient; *SE*—standard error; *LLCI*—lower bound confidence interval; *ULCI*—upper bound confidence interval.

## Data Availability

The data can be made available from the corresponding author upon reasonable request.
